# Bioinformatics Research on Drug Sensitivity Prediction

**DOI:** 10.3389/fphar.2021.799712

**Published:** 2021-12-09

**Authors:** Yaojia Chen, Liran Juan, Xiao Lv, Lei Shi

**Affiliations:** ^1^ Yangtze Delta Region Institute (Quzhou), University of Electronic Science and Technology of China, Quzhou, China; ^2^ School of Life Science and Technology, Harbin Institute of Technology, Harbin, China; ^3^ Beidahuang Industry Group General Hospital, Harbin, China; ^4^ Department of Spine Surgery Changzheng Hospital, Naval Medical University, Shanghai, China

**Keywords:** drug sensitivity, database, machine learning, deep learning, anti-cancer

## Abstract

Modeling-based anti-cancer drug sensitivity prediction has been extensively studied in recent years. While most drug sensitivity prediction models only use gene expression data, the remarkable impacts of gene mutation, methylation, and copy number variation on drug sensitivity are neglected. Drug sensitivity prediction can both help protect patients from some adverse drug reactions and improve the efficacy of treatment. Genomics data are extremely useful for drug sensitivity prediction task. This article reviews the role of drug sensitivity prediction, describes a variety of methods for predicting drug sensitivity. Moreover, the research significance of drug sensitivity prediction, as well as existing problems are well discussed.

## 1 Introduction

With the significant technological advancements, a variety of modalities have been developed to predict the sensitivity of tumors to anti-cancer drugs, which can improve drug efficacy and reduce adverse effects and the financial burden of treatment on patient. The sensitivity of tumors to anti-cancer drugs can be assessed by using patient cell lines, which can facilitate the use of synergistic regimens (Liu et al., 2016). Unfortunately, this process requires substantial time and carries a high risk (Hanna, 2006; Russo, 2015; Cheng et al., 2019; Zhuang et al., 2020). Moreover, tumor drug resistance is another critical problem in the research and development of anti-cancer drugs and the medical field (Liu et al., 2020; Zhang et al., 2020a). It is well known that traditional cancer treatment approaches mainly aim to eradicate rapidly proliferating tumor cells (Restifo et al., 2016; Cheng et al., 2018; O’Donnell et al., 2018; Liu and Chen, 2020; Liu et al., 2021a). However, existing evidence illustrates that tumor cell subgroups can survive by resisting treatment through resistance mechanism, and these cells will finally evolve into drug-resistant tumor cells. It is challenging to elucidate the mechanisms by which tumors acquire drug resistance, predict the evolution of drug-resistant tumors, and determine appropriate strategies to eliminate recalcitrant cells. In addition, the identification of mutations that increase sensitivity to anti-cancer drugs and formulation of appropriate treatment plans for patient groups with specific genomic mutations have essential roles in the development of targeted therapies and achievement of precision treatment for human cancer. However, the traditional strategy for predicting drug sensitivity based on the similarity with known mutations has limitations (Carr et al., 2016; Jennifer et al., 2016; Schmitt et al., 2016; Li et al., 2017; Song et al., 2020; Qi et al., 2021). Meanwhile, the large amount of data resources related to markers of anti-cancer drug sensitivity need to be integrated. Most importantly, among the cancers, some are transmittable, thus triggering panic. Therefore, therapeutic drugs are urgently needed that can stop cancer transmission. The transmission of drug-resistant strains is an extremely serious major public health issue. Meanwhile, drug-resistant mutant strains of HIV-1 have hampered anti-viral treatment. It is necessary to study the mutation and subtype characteristics of HIV-1 recombinants, evolutionary principles, and drug resistance to develop vaccines and related drugs and implement preventative and control measures for AIDS (Castro-Nallar et al., 2012; Hemelaar, 2012; Shaw and Hunter, 2012).

**FIGURE 1:**
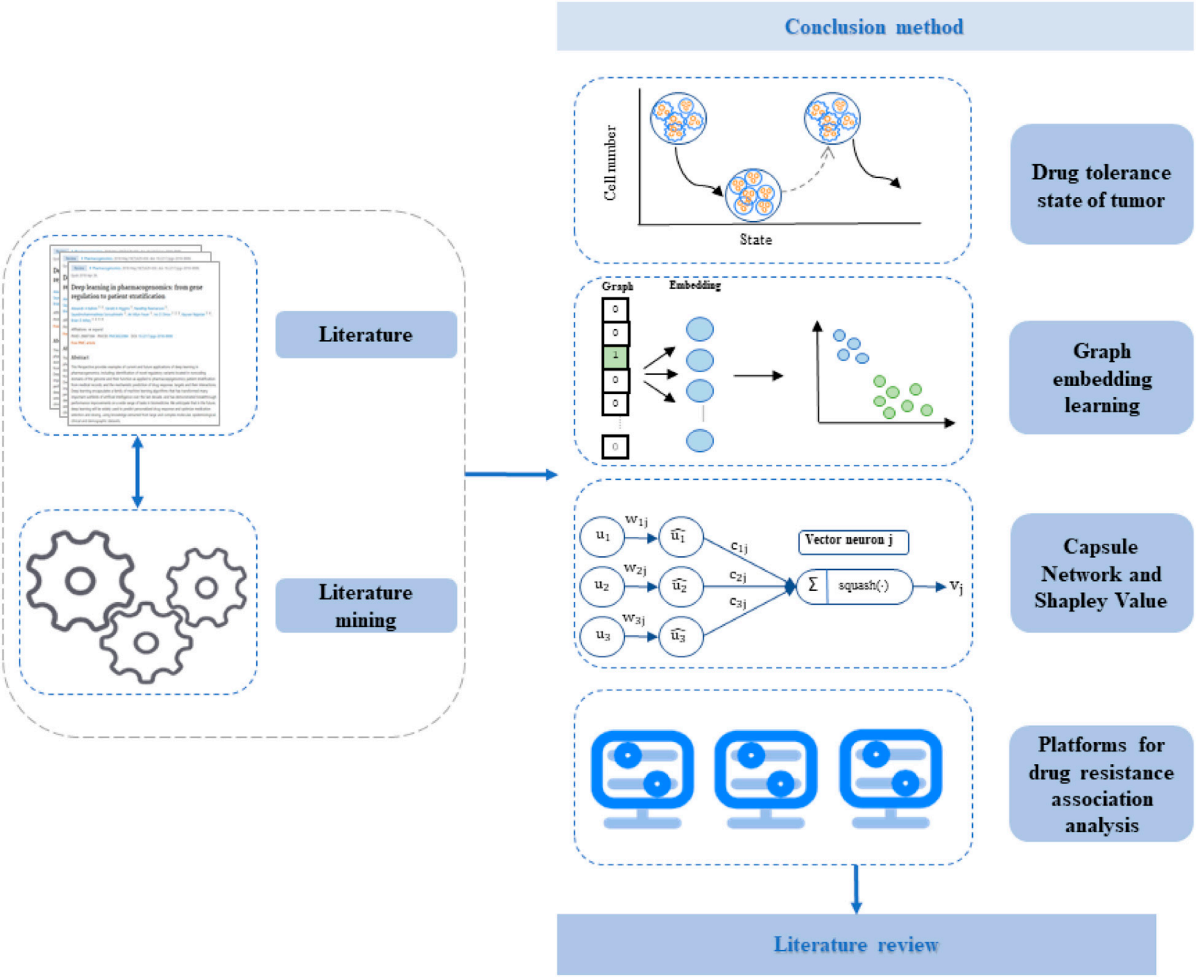
Schematic of the study.

This review focuses on several strategies related to drug sensitivity analysis. We first discuss the types of evolutionary models of drug resistance studies on drug-tolerant tumor cells after treatment. Meanwhile, we describe the process of models, including the construction of a time-series biological network and the evolution prediction of the tumor drug resistance state *via* k-means++ clustering, random walk, and other machine learning methods (Oxnard and Geoffrey, 2016; Hangauer et al., 2017; Recasens and Munoz, 2019; Yu et al., 2020a; Cheng et al., 2021). Second, we describe the strategy of sensitivity prediction of tumors to anti-cancer drugs using graph representation learning. This strategy can explain the mechanism by which cancer develops, and most importantly provide reliable evidence for cancer treatment to promote the development of bioinformatics. Third, we discuss the strategy of developing a collaborative drug sensitivity analysis platform that can provide specific cancer cell lines with optimal stimulatory or inhibitory candidate drug molecules. This strategy provides new technical solutions for the development of anti-cancer drugs and overcomes the insufficiency of deep learning modeling methods for analyzing anti-cancer drug sensitivity (Jaiswal et al., 2018; Zhao et al., 2018; Azad et al., 2019; Cheng et al., 2020; An and Yu, 2021; Shang et al., 2021). The fourth strategy mainly aims to establish platforms for drug resistance association analysis to reduce the blind use of drug-resistant HIV strains and improve the effectiveness of AIDS treatment. There is an urgent need to develop drugs for both AIDS and cancer to limit deleterious effects in patients. Therefore, it is necessary to review bioinformatics research into drug sensitivity prediction. The outline of the essay is provided in Figure 1.

## 2 Methods of Research on Drug Sensitivity

### 2.1 Evolutionary Model Based on Drug Resistance

#### 2.1.1 Introduction to Methods

This type of researches mainly aimed to explore the drug resistance mechanisms of various anti-cancer drugs and the evolutionary direction of the drug resistance state. They target the characteristics of drug-resistant tumors during treatment through machine learning and deep learning methods, as well as the use of high-throughput pharmaceutical informatics data. Scientists generally conduct research from four aspects. 1) The first aspect is mainly concerned with the analysis of different drug resistance mechanisms arising from pan-cancer and tumor drug resistance based on the different tumor cell lines after treatment. 2) The second is mainly focused on the construction of a prediction model for the evolution of tumor drug resistance. In this part, gene mutations in various cell lines can be added to the prediction model according to the mutation frequency of the gene as the evolutionary condition of resistance. 3) The third aspect is mainly concerned with the design of drug application strategies that interfere with the tumor drug tolerance state. This means that the treatment plan can be devised according to genes affected by existing drugs and the classification of anti-tumor action principles. 4) The fourth aspect of methods mainly focus on the verification of the predicted medication plan through gene chip and cell experiments. Finally, the relevant interference drug plan can be developed using the prediction model, and then gene chip detection can be used for comparisons with the results obtained from the prediction model to verify the accuracy of the model.

#### 2.1.2 Introduction to the Process of This Method

**FIGURE 2:**
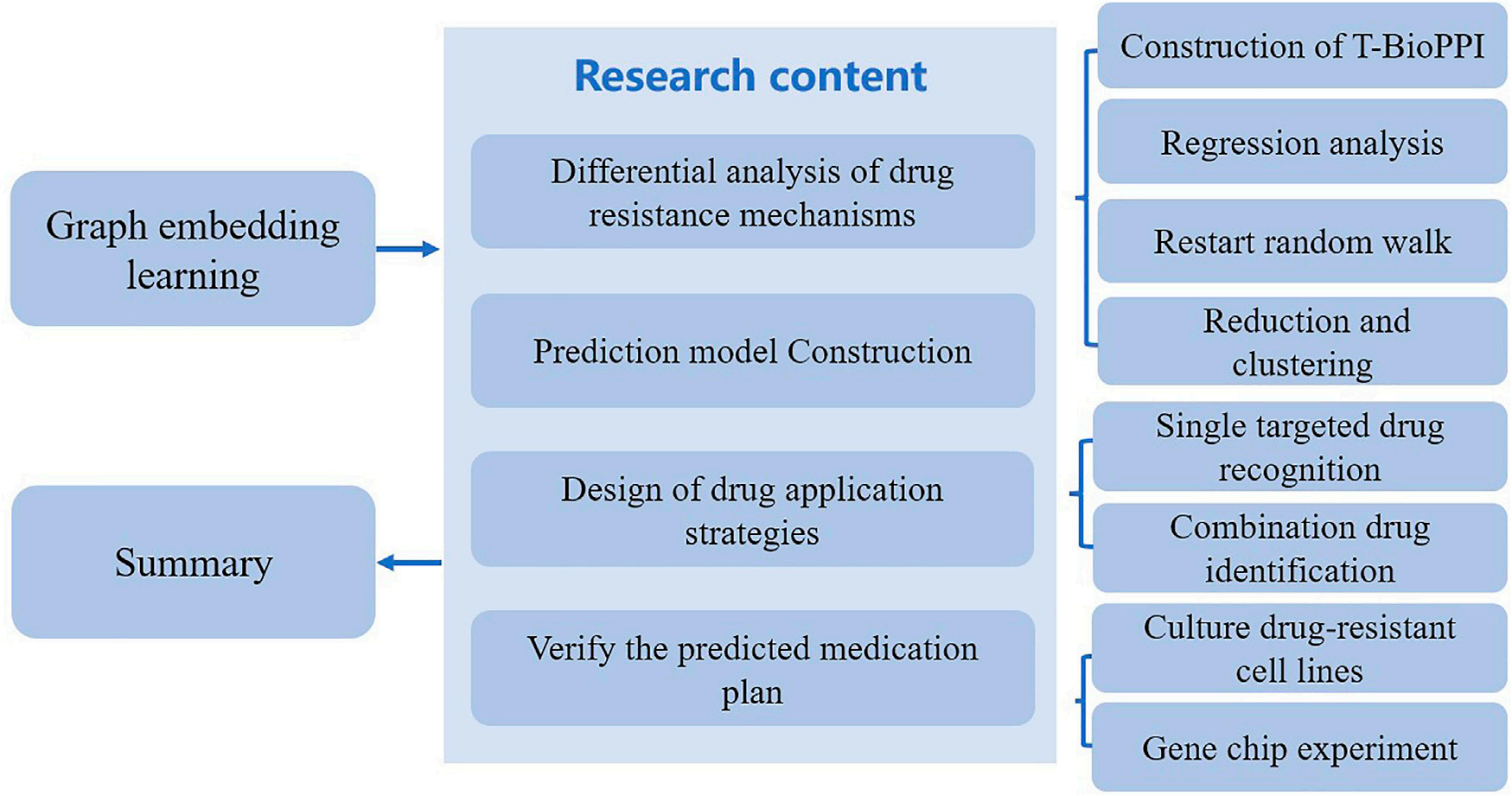
Diagram of the research content on the evolution model of drug resistance.

In recent years, the drug resistance state of tumors, regarded as an important process in the evolution of tumor resistance, has been well studied by using a variety of machine learning methods. This type of methods analyze the drug resistance mechanism activated by tumor cell lines in different drug resistance state. They mainly consist of four steps. 1) The first step is to analyze the drug resistance mechanism produced from the tumor drug resistance state. This step requires the construction of a time-series biological network (T-BioPPI). T-BioPPI integrates multiple database biological association networks to form a relatively comprehensive biological protein interaction network of BioPPI, in which the LINCS database provides tumor cell line gene expression profile data at three time points. The data at these time points are analyzed for differentially expressed genes. Each gene is expressed as a different node between time layers in the biological network. At this point, the gene interaction networks at various time points are linked together to construct a large time-series biological network, thus identifying the important genes in the biological network through regression analysis. Because each drug has a relatively fixed target in the cells, the key lies in the action time of the drug. Using the drug dose as an independent variable and gene expression as a dependent variable, regression analysis of gene expression over time in tumor cells following drug treatment can be performed. Following this step is an initiated step to map the genes of each tumor cell line to T-BioPPI. Then, by restarting the random walk, an analysis of walking from the nodes in the 0-h network to the 24-h network can be performed. This is followed by dimensionality reduction and k-means++ clustering. The evolution prediction model is the classification result of tumor cell lines obtained after clustering. 2) Second, a deep learning model is established for predicting the evolution of the tumor drug resistance state. In general, long short-term memory networks are applied to assess the evolution of tumor drug resistance through ordered sequences of gene expression changes. During this step, the classified cell line data are used as the basis for long short-term memory model construction, and the gene expression profile data of similar tumor cell lines are used as the training set for deep learning data. 3) The third step is to design drug application strategies that interfere with tumor drug resistance to cover single-target drug identification and combination drug identification. 4) The fourth step is to verify the predicted drug resistance evolution model and medication plan through cytology and gene chip experiments. Three main sub-steps are involved. The first sub-step is the cultivation of drug-resistant cell lines, the second sub-step involves gene chip experiments, and the last sub-step is the cell proliferation inhibition test. Figure 2 presents a reference for the drug resistance evolution model based on the tumor drug resistance state and the general process of the drug administration strategy.

### 2.2 Graph Embedding

**FIGURE 3:**
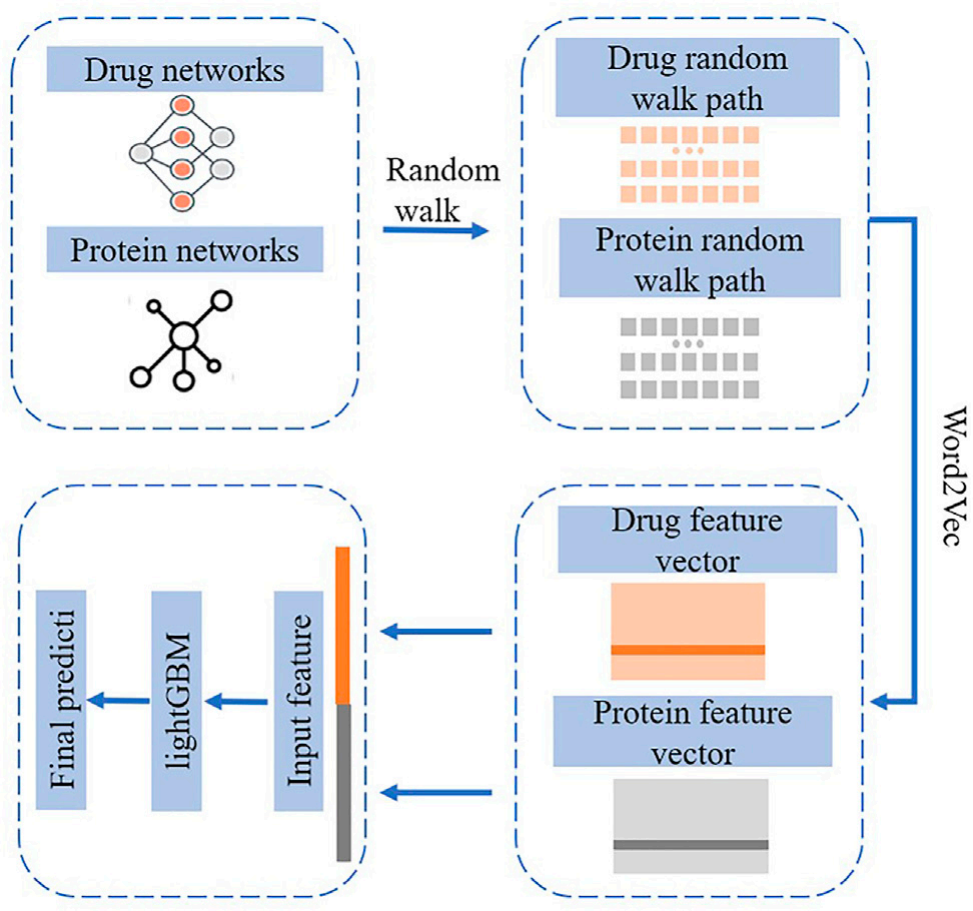
Flowchart of graph embedding-based algorithm NEDTP.

Computational theory tools such as graph representation learning are widely used to establish standard data sets and online databases for anti-cancer drug sensitivity mutation data using. Then analysis models are established to conduct in-depth research and exploration on anti-cancer drug sensitivity mutation prediction methods. The project mainly studied the following aspects. 1) First, the construction of anti-cancer drug sensitivity mutation databases. After collecting anti-cancer drug sensitivity mutation data from multiple cancer genome projects, a document classifier for cancer-related mutations can be constructed by using machine learning text mining technology. The classifier intends to facilitate access to the literature about cancer–mutation–drug information in the PubMed database. After obtaining these related documents, professional personnel can collect and annotate the relevant anti-cancer drug sensitivity mutation information. When mutation data information is obtained for the first time, standard tools are employed to organize the information annotations of each entry into a standard format. Then, the obtained original data sets are integrated with the source of literature mining. Finally, a user-friendly anti-cancer drug sensitivity mutation database web interface based on the Browser/Server model is developed, which is open for users to view and download data. 2) The second aspect focuses on research on the prediction method of drug sensitivity markers. According to the characteristics of drug sensitivity-related mutations, known anti-cancer drug sensitivity mutation data is sorted and preprocessed. Then, existing feature quantification methods are collected and well analyzed. Meanwhile, wild-type and mutant DNA sequences are used for feature quantification to permit the information before and after the appearance of the mutation, which can further ensure the reliability of the results. The background network is obtained by calculating the mutation–mutation similarity. 3) Third, a drug–drug network is extracted from the research on the prediction method of mutation–drug interaction pairs. Then, a multi-source heterogeneous drug interaction network is established through techniques such as similar network fusion. 4) Finally, the graph representation learning method is adopted to predict the relationship of the mutation–drug interaction pair and develop corresponding prediction software and an online prediction platform. The flowchart of graph embedding-based algorithm NEDTP is shown in Figure 3.

### 2.3 Capsule Network and Shapley Value Method

Deep learning has shown impressive performance in many tasks (Jiang et al., 2013; Guo et al., 2020; Jin et al., 2020; Tao et al., 2020; Yu et al., 2020b; Zhang et al., 2020b; Zhao et al., 2020; Jin et al., 2021; Liu et al., 2021b; Lv et al., 2021; Su et al., 2021; Wang et al., 2021a; Xu et al., 2021; Yu et al., 2021). This deep model-based strategy intends to build a deep feed-forward network and drug fingerprint encoding method to obtain the disease cell lines and drug quantitative characteristics. To identify the direct correlation between drug groups and disease-specific gene expression profiles, this project adopted the capsule network and the encoder–decoder model of the attention mechanism to predict the sensitivity of cancer cell lines to single and combination drug regimens. Capsule network is an improved convolutional neural network, which loads the information of feature states learned in the network into capsules in the form of vectors. The capsule preserves precise information about position and posture, making the visual entity locally invariant. While traditional deep learning methods output as a single scalar on a single neuron, and realize the invariance of perspective through maximum pooling method, it loses a lot of valuable information and fails to take into account the relative spatial relationship between coding features. The capsule network can learn the posture information of different cells from the cancer cell line, and convert the information that might be missed by the traditional CNN network into high-level features, which can be used to predict the sensitivity of drugs to the cancer cell line. The contribution/inhibition relationship between drug groups for specific diseases was obtained using the Shapley value method of cooperative game theory to analyze the convolutional neural network model (Aumann and Shapley, 1971; Karim et al., 2019; Cai et al., 2020a; Cai et al., 2020b; Mo et al., 2020), as well as through calculation and comparative analysis of the marginal contributions between drug groups. The project mainly studied three points: 1) Feature quantification methods oriented at the prior knowledge of genomics and drug targeting information to construct a deep feed-forward network and encode drug fingerprints based on drug targeting relationships. 2) Designing a deep learning model adapted to gene expression profile and drug gene data. First a network structure is built to analyze the basic structural association relationship between drug groups and gene expression profiles. And the capsule network is used to extract the characteristics of the cancer cell line and the drug itself. Moreover, the encoder–decoder model of the attention mechanism is adopted for the fusion of heterogeneous features. 3) Constructing a gene expression profile–drug group network by cooperative game model. Then the gene-drug group network is applied to calculate the enhancement/inhibition degree of the drug fingerprint and identify the set with obvious enhancement/inhibition effects in the drug group. This type of method considers both the enhancement/inhibition relationship between drug combinations. It first integrates the drug combination data from various sources, then extracts the enhancement/inhibition relationship combinations of different drug combinations, and finally predicts the sensitivity of drug combination with machine learning algorithms based on the different feature combinations.

### 2.4 Drug Resistance Association Analysis

**FIGURE 4:**
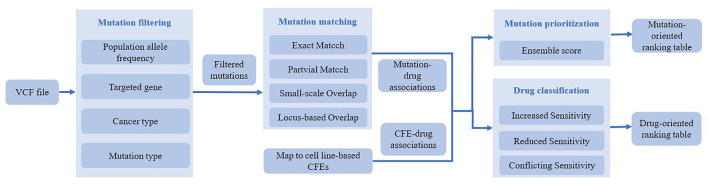
Flowchart of representative platform for drug resistance mutations prediction.

The strategy mainly aims to establish a recombinant strain drug resistance analysis platform, and verify the hypothesis related to recombinant drug resistance by targeting circulating recombinant forms (CRFs) (Ru et al., 2020; Wang et al., 2020a). Data related to drug resistance can be obtained through appropriate and efficient data mining methods. By combining SeqFeatR and Bayesian factor methods, complex hierarchical models can be used to quantify drug combinations (Plummer, 2003; Bettina et al., 2016; Zhao et al., 2019; Hu et al., 2020; Zeng et al., 2020a; Zeng et al., 2020b; Hu et al., 2021a; Hu et al., 2021b; Song et al., 2021). Meanwhile, reliable associations can be identified for recombinant HIV-1 for application in anti-viral therapy based on the link between base substitutions in viral sequences and the viral genomic background. The direct coupling analysis method is used to predict the interaction between the associated mutations in the protein and analyze the nearest neighbor between the sites associated with drug resistance. The main research contents are as follows: 1) establish a drug resistance association analysis platform for HIV-1 CRFs and 2) propose and verify that the HIV-1 CRFs are related to drug resistance mutations. This method mainly aims to establish a recombinant HIV-1 drug resistance analysis platform and spread it to other recombinant pandemic areas across the globe. It initially involves data and model inference. In this project, HIV-1 pol serves as the research object, and model inference is achieved through JRip of the RWeka software package of the R system, which makes fast rule inference on the aforementioned three sets of data by adopting the RIPPER algorithm. Then, reliability verification of the model inference (leave-one-out classification verification) is conducted to obtain the statistical evaluation of the rule inference results (Zeng et al., 2018; Zeng et al., 2019; Dao et al., 2020; Fu et al., 2020; Zulfiqar et al., 2021). Finally, the sequence characteristics of recombinant HIV-1, recombinant drug-related patterns, and recombinant characteristic drug resistance patterns will be obtained. In general, this method performs drug resistance information interpretation and correlation analysis of HIV-1 recombinant characteristic drug resistance mutations, and then performs computer modeling and construction verification. The flowchart of representative platform for drug resistance mutations prediction is shown in Figure 4.

### 2.5 Summary

This chapter mainly discusses the significance and indispensability of drug sensitivity in bioinformatics research by introducing different methods. A persistent problem has arisen in the research and development of anti-cancer drugs and in the medical and health fields, namely the issue of tumor resistance. Methods reviewed in this study can assist with the prediction of drug sensitivity. They encompass oncology, pharmacy, and computer science, and strategies to predict tumor resistance, design rational drug strategies, and construct computer models were principally covered. These methods have promoted the research and development of bioinformatic fields such as computational methodology and algorithm design. Moreover, research reviewed in this study can be directly applied to anti-cancer precision medicine, new drug identification, and other systems. They have exhibited broad market application prospects and a further possibility to improve the effectiveness of AIDS treatment and lower the cost of its prevention and control.

## 3 Literature Contribution

**FIGURE 5:**
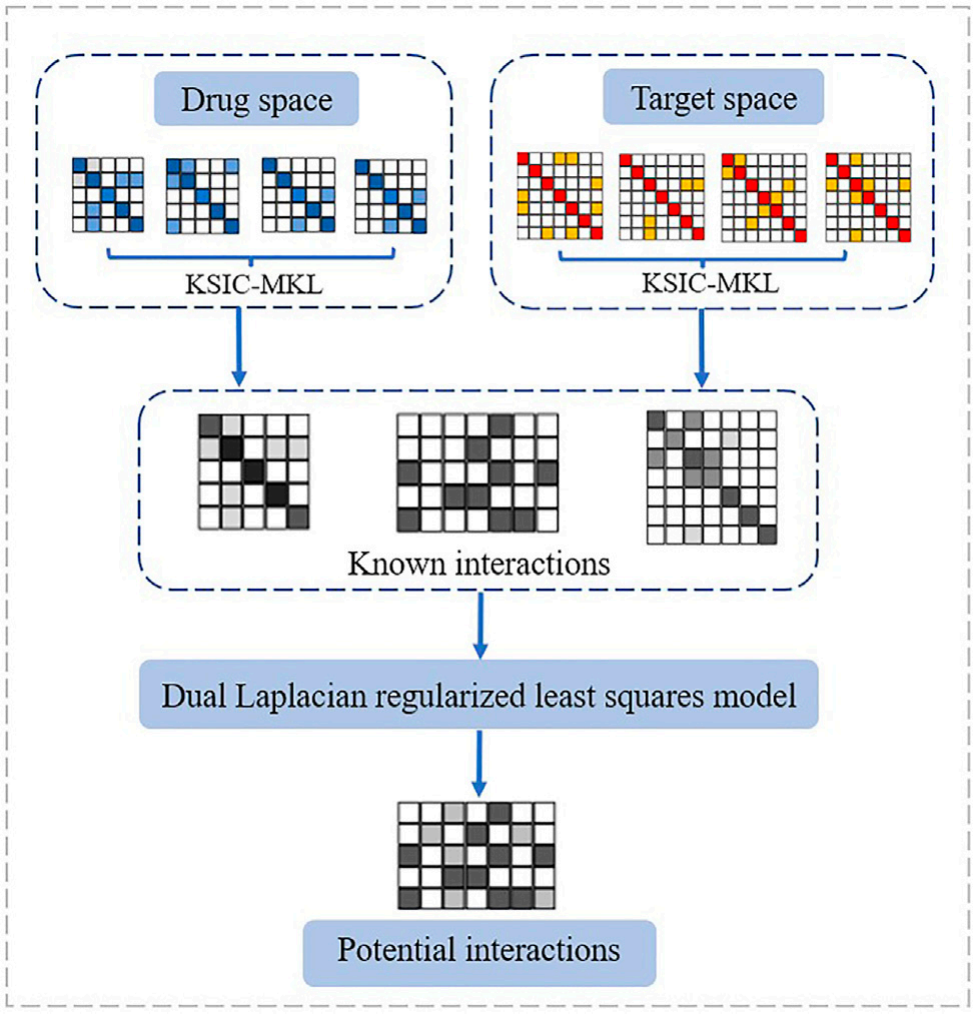
Flowchart of DLapRLS.

Tumor drug tolerance is an important process in the evolution of tumor drug resistance, and the drug resistance mechanism activated by the tumor in the drug resistance state remains unclear. Bioinformatic analysis and research on mutations associated with anti-cancer drug sensitivity are expanding. Hopefully, the methods reviewed in this study will contribute to overcoming existing problems. They analyzed the drug resistance mechanism of tumor cell lines in a drug-resistant state based on a variety of machine learning (Wei et al., 2014; Wei et al., 2017a; Wei et al., 2017b; Ding et al., 2020a; Ding et al., 2020b; Wang et al., 2020b; Wang et al., 2021b) and deep learning methods to study the gene expression profiles of a large number of drug-resistant tumor cells (Lv et al., 2019; Su et al., 2019; He et al., 2020; Li et al., 2020; Peng et al., 2020; Su et al., 2020; Zhang et al., 2020c; Cui et al., 2021). The flowchart of a representative method DLapRLS is shown in Figure 5. The established prediction model provides a new strategy for future research on tumor drug resistance. Cell experiments are also applied to block the evolution of drug resistance in the tumor drug resistance state by using single drugs and combination regimens. A new random walk-based graph representation learning algorithm was proposed to the predict of anti-cancer drug sensitivity mutation data. It incorporates gene–drug interaction network information into the node representation of mutation–mutation networks for the comprehensive and systematic command of the inherent properties of such mutations. Moreover, a mutation–drug network graph representation algorithm with multi-source heterogeneous information was developed to predict mutations associated with anti-cancer drug sensitivity and sensitivity/resistance to specific drugs. Meanwhile, a network prediction platform available for researchers was also developed (Zhang et al., 2020a). An exploratory method based on the Shapley value of cooperative game theory was proposed to analyze the convolutional neural network model. Through the differential analysis of the contributions of monotherapies and synergistic drug combinations in specific disease cell lines, candidate drug group collection of the enhancement/inhibition relation in drug components was obtained. Further application and promotion of the drug resistance association analysis platform can provide strategies for controlling the spread of circulating recombinant drug-resistant HIV-1 strain. At the same time, it can also help reduce the blind use of drugs against circulating recombinant drug-resistant HIV-1 strain, improve the treatment effectiveness, and lower the cost of prevention and control of AIDS.

## 4 Conclusion

In recent years, anti-cancer drugs have consistently been the focus of new drug development. Methods that can accurately predict drug sensitivity are urgently needed to facilitate drug development and disease prevention in the field of biomedical health. In this study, a comprehensive review was provided concerning the analysis of the drug resistance mechanisms of tumor cell lines in the drug-resistant state by using a variety of machine learning methods. The two-step cancer–mutation–drug triad prediction is achieved through text mining technology based on machine learning. It has laid solid foundations for the subsequent construction and update of the drug sensitivity mutation database through a combination of manual annotations. The wide distribution of HIV-1 recombinant types and the formation of drug-resistant strains will facilitate our study of recombinant characteristic drug resistance patterns and drug resistance associations. The aforementioned summary indicates the necessity of the current review.
